# AFFORDABLE SENIOR HOUSING AND HEALTH AMONG LOW-INCOME OLDER IMMIGRANTS: THE ROLE OF SERVICE USES

**DOI:** 10.1093/geroni/igz038.3444

**Published:** 2019-11-08

**Authors:** Jihye Baek, Oejin Shin, Sojung Park, BoRin Kim, Byeongju Ryu

**Affiliations:** 1 Brown School, Washington University in St. Louis, Saint Louis, Missouri, United States; 2 University of Illinois at Urbana-Champaign, Urbana-Champaign, Illinois, United States; 3 University of New Hampshire, Durham, New Hampshire, United States

## Abstract

Older immigrants in affordable senior housing face a unique set of challenges due to their demographic, social, economic, and cultural diversity. Existing knowledge about health among this unique but increasing aging subgroup population is extremely limited. Focusing on older immigrants subgroups (Asian and Russian older adults) in affordable senior housing in St.Louis, MO, this study aimed to examine to what extent different ethnic minority elders’ health varies by their uses of services available in the housing. Data came from the survey interviews at a subsidized independent senior housing in St. Louis (n=136). Hierarchical multiple regressions were used to examine ethnic differences in self-rated health and the role of services for the health of low-income ethnic minor elders in senior housing. Compared to the non-immigrants (White/African older adults), Asian (b=0.67, p<.05) and Russian residents (b=0.89, p<.05) were likely to have lower self-rated health. Interestingly, for both ethnic groups, they report a better self-rated health when they use supportive daily service (i.e. e.g. meal delivery, transportation, housekeeping and others) (b= -0.84, p<.05 for Asian, b=- 0.90, p<.05.for Russian) and social service (e.g. recreational, wellness, and exercise programs) (b= -0.73, p<.05 for Asian, b=- 0.83, p<.05.for Russian). Our findings point to an important role of services for the health of low-income ethnic minor elders in senior housing. As the first attempt to examine services that explicitly focus on ethnic minority elders, our study provides meaningful implications for future research on the health and service needs for older immigrant populations in senior housing.

